# Predicting health services utilization using a score of perceived barriers to medical care: evidence from rural Senegal

**DOI:** 10.1186/s12913-023-09192-2

**Published:** 2023-03-16

**Authors:** Marion Coste, Marwân-al-Qays Bousmah

**Affiliations:** 1 Aix Marseille University, CNRS, AMSE, Marseille, France; 2 Aix Marseille University, INSERM, IRD, SESSTIM, Sciences Economiques & Sociales de la Santé & Traitement de l’Information Médicale, ISSPAM, Marseille, France; 3 Université Paris Cité, IRD, Inserm, Ceped, Paris, F-75006 France

**Keywords:** Healthcare access, Perceived barriers, Primary care, Universal health coverage (UHC), Rural, Senegal, Sub-Saharan Africa

## Abstract

**Background:**

Ensuring access to healthcare services is a key element to achieving the Sustainable Development Goal 3 of “promoting healthy lives and well-being for all” through Universal Health Coverage (UHC). However, in the context of low- and middle-income countries, most studies focused on financial protection measured through catastrophic health expenditures (CHE), or on health services utilization among specific populations exhibiting health needs (such as pregnancy or recent sickness).

**Methods:**

This study aims at building an individual score of perceived barriers to medical care (PBMC) in order to predict primary care utilization (or non-utilization). We estimate the score on six items: (1) knowing where to go, (2) getting permission, (3) having money, (4) distance to the facility, (5) finding transport, and (6) not wanting to go alone, using individual data from 1787 adult participants living in rural Senegal. We build the score via a stepwise descendent explanatory factor analysis (EFA), and assess its internal consistency. Finally, we assess the construct validity of the factor-based score by testing its association (univariate regressions) with a wide range of variables on determinants of healthcare-seeking, and evaluate its predictive validity for primary care utilization.

**Results:**

EFA yields a one-dimensional score combining four items with a 0.7 Cronbach’s alpha indicating good internal consistency. The score is strongly associated—p-values significant at the 5% level—with determinants of healthcare-seeking (including, but not limited to, sex, education, marital status, poverty, and distance to the health facility). Additionally, the score can predict non-utilization of primary care at the household level, utilization and non-utilization of primary care following an individual’s episode of illness, and utilization of primary care during pregnancy and birth. These results are robust to the use of a different dataset.

**Conclusion:**

As a valid, sensitive, and easily documented individual-level indicator, the PBMC score can be a complement to regional or national level health services coverage to measure health services access and predict utilization. At the individual or household level, the PBMC score can also be combined with conventional metrics of financial risk protection such as CHE to comprehensively document deficits in, and progress towards UHC.

**Supplementary Information:**

The online version contains supplementary material available at 10.1186/s12913-023-09192-2.

## Background

Achieving universal access to healthcare services is a key element to the Sustainable Development Goal 3 of “ensur[*ing*] healthy lives and promot[*ing*] well-being for all at all ages” [1]. Specifically, target 3.8 sets to “achieve universal health coverage (UHC), including financial risk protection, access to quality essential health-care services and access to safe, effective, quality and affordable essential medicines and vaccines for all” (ibid.). The standard metrics for measuring progress towards the financial risk protection aspect of UHC are catastrophic health expenditures (CHE) [2], identifying whether out-of-pocket (OOP) health expenditures represent a “catastrophic” share of the overall household expenditures, usually set at 40% [2–4], or impoverishing health expenditures, which document whether the household’s falling below the poverty line is attributable to health expenditures [5]. These metrics can be easily computed from widely available household surveys.

A recent comprehensive assessment of UHC progress combined CHE prevalence with a measure of service coverage capturing both prevention and treatment indicators at the country level [6]. Service coverage is meant to document the aspects of access which are part of UHC and might be at odds with CHE, especially in the context of low- and middle-income countries (LMICs) where lower OOP might reflect the lower quality of health services [7, 8], unmet health needs [9], or even a younger, healthier population [10]. Indeed, Wagstaff and colleagues found an association between low incidence of CHE and low service coverage in LMICs.

At the population level, access to quality health services is usually measured through health services utilization [11], often within specific populations exhibiting health needs, e.g., children’s immunization records, women with a recent pregnancy, or individuals having experienced a recent or chronic illness. It involves heavy data collection processes and long interviews focusing on specific events in a given timeframe (e.g., two years for recent pregnancy and birth, 12 months for inpatient visits, etc.).

In LMICs, the literature has specifically investigated women’s self-reported barriers to seeking medical care [12, 13], which are collected as part of the Demographic and Health Surveys (DHS) [14]. These questions record perceptions on both the financial (possession of, or perceived ability to obtain monetary resources) and the geographic accessibility (distance and transportation means) as well as barriers pertaining to cultural and social norms (i.e., concerns about obtaining permission and going alone)—thereby covering a wide range of elements which have been identified as determinants to healthcare seeking and health services utilization [15–19].

Existing studies have documented an association between reporting at least one significant barrier and lower maternal and prenatal health services utilization [20–22]. A 2012 study combined socioeconomic, geographical, and psychosocial barriers from the 2003 DHS in Burkina Faso to create a tri-dimensional score of women’s perceived ability to overcome barriers to healthcare seeking [23] and validated the score in relation to a select number of socio-demographic variables (specifically age, education level, poverty status and rural versus urban living) without investigating associations with the utilization of maternal or child services. In addition, all these studies solely focused on women.

This study seeks to elaborate, and validate a synthetic measure of perceived access to healthcare in both men and women, in the context of LMICs. We investigate whether items on perceived barriers to medical care can be combined into a score, and assess both the score’s construct validity (with respect to documented determinants of healthcare seeking), and predictive validity (with respect to primary care utilization).

## Methods

### Study setting and design

We employed individual data from the CMUtuelleS survey, a cross-sectional survey conducted in 2019–2020 among 1787 residents of the Niakhar Health and Demographic Surveillance System (HDSS) in rural Senegal [24]. In Senegal, health dispensaries are the first level of permanent health facilities: they are run by a chief nurse, alongside an assistant nurse, a midwife, and community healthcare workers. Health dispensaries are the first point of entry into the healthcare system and, in the absence of complications, it is also where pre-natal consultations and birth take place. The Niakhar HDSS counts three semi-urban villages, Diohine, Toucar, and Ngayokheme, where health dispensaries are located. The second level of health facilities is health centers with at least one physician working full-time. Inhabitants of the Niakhar HDSS of the Ngayokheme municipality go to the Niakhar health center located in the town of Niakhar, just outside of the HDSS area. People living in the Diarère municipality go to the health center located in the city of Fatick– 10 km away by paved road, where the regional referral hospital is also implanted. Municipalities also determine affiliation to either the Ngayokheme or the Diarère community-based insurance (CBHI) offices.

The CMUtuelleS survey aimed at characterizing the implementation of CBHI schemes among voluntary subscribers who paid the full fee, and beneficiaries of the national cash transfer program for poor households (BSF, “B*ourse de Sécurité Familale*”), whose subscription to the CBHI was supposed to be subsidized [25, 26]. Accordingly, both the subscriber/head of household and their partner were interviewed among three groups: voluntary subscribers (n = 285), BSF recipients (n = 176), and non-enrolled in a CHBI scheme (n = 1326).

### DHS-based items on barriers to medical care

In an adaptation from the 2008 Demographic and Health Survey (DHS)’s woman’s questionnaire^1^, both male and female participants were asked “*When you are sick, or you want to get medical advice or treatment is any of the following i) not a problem, ii) a small problem, or iii) a big problem*:

1. *knowing where to go?*
2. *getting permission to go?*
3. *getting the money to pay?*
4. *the distance to the health facility?*
5. *having to take transport?*
6. *not wanting to go alone?”.*

### Data

The CMUtuelleS dataset contained rich self-reported micro-level data on the individuals and their households. In addition to standard socio-demographic variables, including, age, education level, sex and marital status, data reported GPS coordinates, which were used to compute distances between the household and the nearest health facility and CBHI office, respectively. The survey recorded the participants’ health insurance status and self-reported health (12-Item Short Form Survey questionnaire [27], chronic illness, and handicap). Additionally, participants reported perceived quality of care at the local healthcare facility, knowledge of community-based health insurance, willingness to pay for health insurance, risk aversion [28], and generalized trust [29]. The survey also extensively quantified the household’s expenditures (including monthly consumption expenditures per adult equivalent, and out-of-pocket health expenditures) and included several measures of poverty (specifically, monetary, food, and subjective poverty). Catastrophic health expenditures were computed following Xu et al. [2].

Finally, the survey documented individual-level primary care utilization following health needs (consultation, self-medication, exams, or hospitalization among participants with an episode of illness in the past two months; prenatal consultations and health facility delivery among women who had a live birth in the past two years), as well as unmet health needs at the household level (having forgone healthcare expenses in the past 12 months). All these variables were defined in Appendix A2 in the Supplementary Material.

### Building the score

After checking for sample adequacy using the Kaiser-Meyer-Olkin measure and the Bartlett test of sphericity [30], the score was built on DHS-based barriers to medical care using stepwise descendant explanatory factor analysis (EFA). Starting with the full 6-item set, each item was removed one at a time to test whether any of the reduced form factor analyses provided a better fit to the data. The number of dimensions to retain was selected following scree plot analysis with a conservative Kaiser criterion of eigenvalues > 1.1 [31]. Factors were rotated to provide a clearer pattern of items loaded on each factor, and only items that contributed to the factors’ dimension (i.e., with factor loadings sufficiently high) were retained to create the final score. The internal consistency of the final set of items was assessed using Cronbach’s alpha [32], and a factor-based score was computed as the average of items.

### Construct validity

Following Nikiema et al. [23], we assessed the construct validity of the factor-based score by testing its association with a series of variables, which were grouped into three main categories: (i) documented determinants of healthcare-seeking, (ii) other potentially associated variables, and (iii) catastrophic health expenditures. We ran univariate regressions of the factor-based score on each variable (logistic, multinomial logistic, and linear regressions for binary, polytomous, and continuous variables, respectively).

### Predictive validity

We assessed the predictive validity of the factor-based score by testing its association with variables on primary care utilization at the household level (foregone consultation or treatment in the past 12 months), among participants with a recent episode of illness (consultation, auto-medication) and among women with a recent history of live birth (birth in a health facility, pre-natal consultations). We ran univariate regressions of the factor-based score on each dependent variable (logistic and Poisson regressions for binary and count variables, respectively). For each univariate regression, we provided both the estimates (odds ratios for logistic regressions and incidence-rate ratios for Poisson regressions) and the predicted values (predicted probabilities for logistic regressions and predicted number of events for Poisson regressions) to fully investigate the predictive power of the score as both types of results are useful and complementary [33]. Predictions for the dependent variables were calculated at three representative values of the factor-based score: 0 (“not a problem” for all potential barriers), 1 (“a small problem” on average), and 2 (“a big problem” for all potential barriers).

### Confirmation in an alternative data set

The score was computed with confirmatory factor analysis (CFA) [34] in the dataset of the ANRS12356 AmBASS survey using the same set of items selected by EFA in the CMUtuelleS dataset. The AmBASS survey was conducted in the Niakhar HDSS in 2018–2019 and featured a sample representative of the general population living in the area [35, 36]. Its dataset contains information on determinants of healthcare-seeking as well as self-reported and observed primary care utilization, which allowed to assess construct and predictive validity of the score in the general population living in the Niakhar HDSS. For sensitivity analysis purpose, the score was also re-built using stepwise descendant EFA to investigate potential differences in the structure of the score (i.e. on the set of items selected by EFA).

In all regressions, standard errors were clustered at the household level to account for intra-household correlation. Regressions were weighted using sampling weights to account for choice-based stratified samples. We used a significance level of p < 0.05. All estimations were performed using Stata version 16 [37].

## Results

### Perceived barriers to medical care

**Fig. 1:**
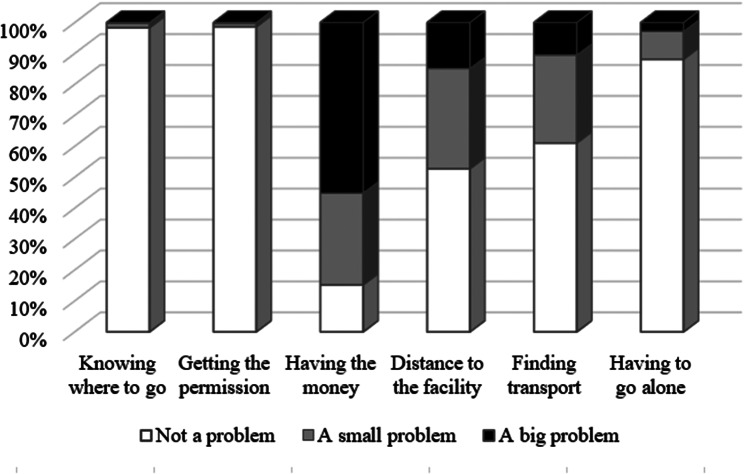
Perceived barriers to medical care (items considered for the score)

Figure 1 presents descriptive results on perceived barriers to medical care. For almost all participants, knowing where to go and getting permission was “not a problem” (98.3% and 98.6% respectively). Having to go alone was “not a problem” either for 88.1% of participants, “a small problem” for about 10%, and “a big problem” for only a small share (2.8%). In contrast, over half of the participants (55.1%) reported that having the money to pay was “a big problem”, with an extra 531 participants declaring it as “a small problem”. Distance to the health facility and finding transport was “not a problem” for a majority of participants (57.2% and 61.1% respectively), “a small problem” for about a third (32.5% and 28.5%), and “a big problem” for 14.8% and 10.5% of participants, respectively.

### Score building

Our sample passed the Bartlett test of sphericity, rejecting the null hypothesis that variables were not inter-correlated (γ²=2080.857(15), p < 0.001), and gave a value for the Kaiser-Meyer-Olkin measure sufficiently large (0.645) to justify running a factor analysis. Stepwise descendant factor analysis suggested that removing the item “knowing where to go” did not significantly reduce the quality of the factor analysis. Subsequent analyses of the score were therefore performed on items (2)-(6). Following EFA and scree plot analysis, only one dimension was retained (2.13 eigenvalue, explaining 42.6% of variations; detailed results were provided in Appendix A3 in the Supplementary Material). Rotations with weights revealed that only items (3)-(6) significantly contributed to dimension one (loadings > 0.4). The 0.7 Cronbach’s alpha of this reduced set indicated very good internal consistency^2^. We, therefore, built a factor-based score with the average of these four items. This score of perceived barriers to medical care (hereafter, PBMC score) was comprised between zero and two, with a mean (standard deviation) value of 0.67 (0.47)—and a 0.5 (0.25-1) median (interquartile range) value. A graph displaying the distribution of the score is presented in Appendix A3.

### Validity

Summary statistics for all variables used are provided in Appendix A4. All univariate regression results are presented in Table 1 (construct validity) and Table 2 (predictive validity). Coefficient estimates (CE) are provided for linear regressions, odds ratios (OR) for logistic regressions, incidence-rate ratios (IRR) for Poisson regressions, and relative-risk ratios (RRR) for multinomial logistic regressions. We also provide graphical representations of the univariate regression results for each of the groups of variables; they are displayed in Appendix A5 in the Supplementary Material.

#### Construct validity

A higher PBMC score was significantly associated (p-value significant at the 5% level) with being a woman, being less formally educated, being unmarried, being poor (whether in terms of monetary, food or subjective poverty, or lower monthly consumption expenditures), being in a smaller household, living further away from the nearest healthcare structure or CBHI office. As an example, a one-unit increase in the PBMC score was associated with the odds of living in a poor household (defined in terms of monetary poverty) increasing by a factor of 1.37. When it comes to distance, a one-point increase in the PBMC score was associated with living 1.32 km further away from the nearest healthcare structure. More specifically, perceiving barriers to healthcare seeking as “not a problem” was associated with living 2.24 km away from the nearest health structure, while perceiving barriers as “a big problem” was associated with living 4.89 km away from the nearest health structure. Age was the only variable not significantly associated with the PBMC score.

**Table 1:** Association of the PBMC score and selected variables (univariate regressions)

Variable group	Dependent variable	Model	Type of estimate	Estimate		p-value
**Determinants of healthcare-seeking**	Had primary education or higher	Logistic	OR	0.62^**^	(0.10)	0.002
	Was a woman	Logistic	OR	1.53^***^	(0.12)	<0.001
	Was in a union	Logistic	OR	0.68^*^	(0.13)	0.042
	Age	Linear	CE	-0.81	(0.92)	0.376
	Was poor (monetary poverty, HH level)	Logistic	OR	1.37^*^	(0.19)	0.023
	Was poor (food poverty, HH level)	Logistic	OR	1.45^**^	(0.20)	0.007
	Was poor (subjective poverty, HH level)	Logistic	OR	1.77^***^	(0.26)	<0.001
	Monthly consumption expenditures per adult equivalent (in CFA)	Linear	CE	-1611.87^*^	(672.56)	0.017
	Number of adult equivalents in the household (HH level)	Linear	CE	-1.12^**^	(0.39)	0.004
	Distance to the nearest healthcare structure (in km)	Linear	CE	1.32^***^	(0.13)	<0.001
	Distance to the nearest CBHI (in km)	Linear	CE	0.52^**^	(0.19)	0.006
**Other potentially-associated variables**	Had an at least fair knowledge of CBHI	Logistic	OR	0.64^**^	(0.09)	0.001
	Health insurance status	Multinomial logistic	RRR (Voluntary)	0.52^***^	(0.09)	<0.001
			RRR (Subsidized)	1.82^**^	(0.34)	0.001
	Willingness to pay for CBHI (in CFA francs)	Linear	CE	-1019.09^***^	(216.71)	<0.001
	Had a chronic illness	Logistic	OR	1.69^*^	(0.37)	0.017
	Had a handicap	Logistic	OR	1.75^*^	(0.46)	0.031
	Had a poorer health	Logistic	OR	1.86^***^	(0.25)	<0.001
	SF-12 Mental Component Summary	Linear	CE	0.35	(0.49)	0.475
	SF-12 Physical Component Summary	Linear	CE	-1.63^*^	(0.65)	0.012
	Perception of healthcare quality	Linear	CE	0.16^***^	(0.03)	<0.001
	Risk tolerance	Linear	CE	-0.61^***^	(0.15)	<0.001
	Generalized trust	Linear	CE	-0.41^**^	(0.13)	0.002
**Catastrophic health expenditures**	Had catastrophic health expenditures, 40% threshold (HH level)	Logistic	OR	1.45	(0.43)	0.203
	Had catastrophic health expenditures, 30% threshold (HH level)	Logistic	OR	1.05	(0.24)	0.844
	Had catastrophic health expenditures, 20% threshold (HH level)	Logistic	OR	1.17	(0.21)	0.370

Notes: *p < 0.05, **p < 0.01, ***p < 0.001. All variables measured at the individual level, unless when HH-level specified. Robust standard errors (clustered at the household level to account for intra-household correlation) in parenthesis. Regressions were weighted using sampling weights to account for choice-based stratified samples. All binary dependent variables were coded as 0 for “no” and 1 for “yes”

Abbreviations: HH=household, OR=odds ratio, CE=coefficient estimate, CBHI=community-based health insurance, RRR=relative-risk ratio

A higher PBMC score was also associated with lower odds of knowing about the CBHI scheme, lower odds of having voluntarily enrolled in a CHBI scheme, and higher odds of benefiting from a subsidized CBHI enrollment through the BSF program. The PBMC score was also negatively associated with the willingness to pay for CBHI schemes. Facing higher barriers to medical care was associated with having a chronic illness, a handicap or disability, and poorer self-assessed health. Interestingly, the PBMC score was tied to physical health (negative association with the SF-12 Physical Component Summary score), but independent of mental health (no association with the SF-12 Mental Component Summary score). Finally, reporting higher barriers to medical care was associated with a lower perception of the quality of local healthcare services, lower risk tolerance, and lower generalized trust.

In contrast, catastrophic health expenditures were not significantly associated with the PBMC score. Note that this result was robust to the use of alternative thresholds of catastrophic health expenditures (namely, out-of-pocket health expenditures ≥ 40%, 30%, and 20% of non-food expenditures, respectively—as displayed in Appendix A5.3 in the Supplementary Material).

#### Predictive validity

**Fig. 2:**
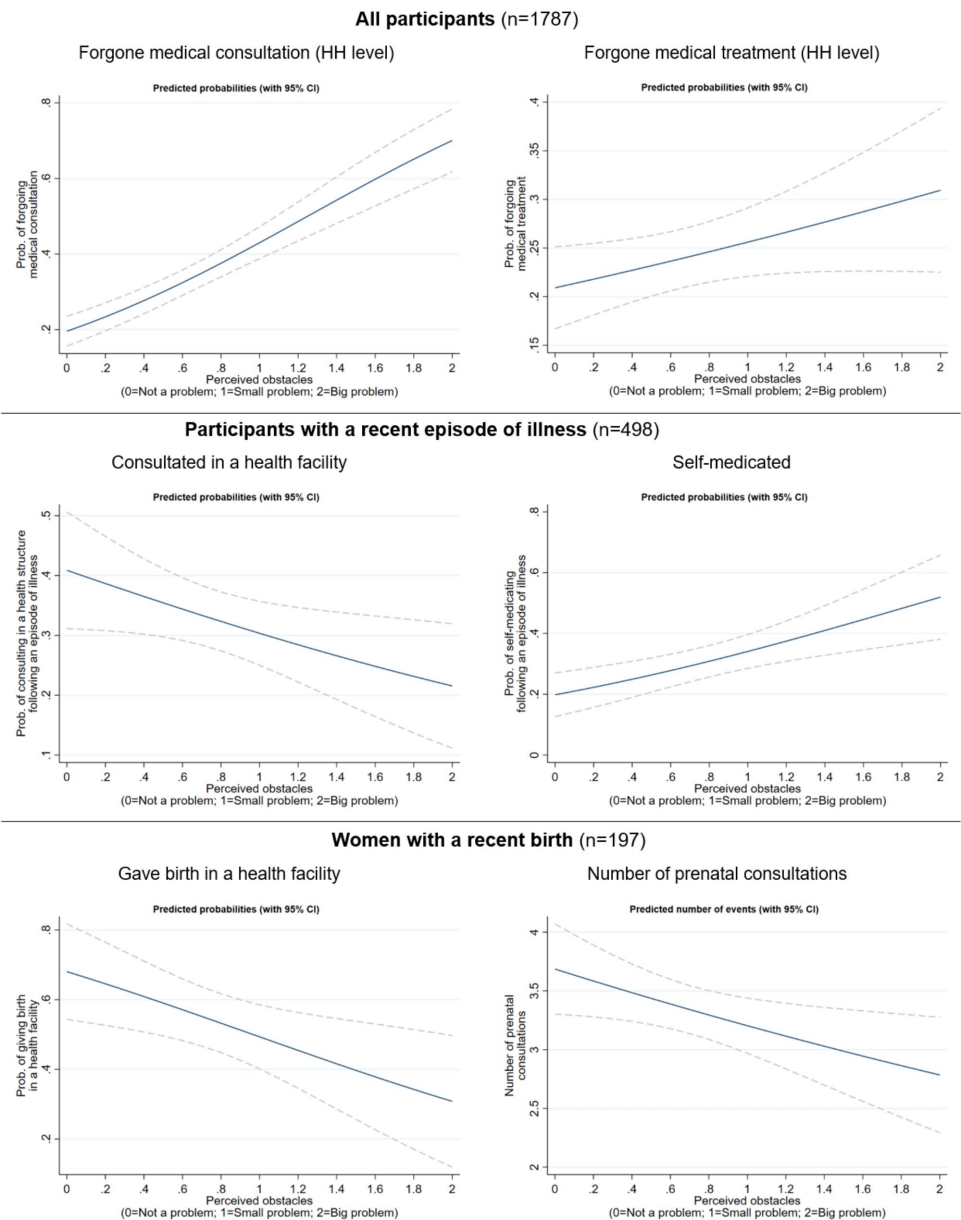
Predicted utilization and non-utilization of primary care across the distribution of the PBMC score. The solid lines are the predicted values (predicted probabilities for logistic models and predicted number of events for the Poisson model), and the dashed lines are 95% confidence intervals

Along with the univariate regression results provided in Table 2; Fig. 2 displayed graphical representations of the predictions of primary care utilization and non-utilization across the distribution of the PBMC score.

The PBMC score was positively associated with the households’ probability of forgoing medical consultation, with a one-unit increase in the score being associated with a 3.10-fold increase in the odds of forgoing medical consultation. In terms of predictions, perceiving all four barriers to healthcare seeking as “not a problem” (i.e. 0 PBMC score) was associated with a 20% predicted probability of foregoing medical consultation, while perceiving all barriers as “a big problem” (a 2 PBMC score) was associated with a 50%-point higher probability (i.e., 70%). This was true to a lesser extent—p-value only significant at the 10% level—for the probability of foregoing medical treatment, with a 10%-point increase in probability from 21% (“not a problem” for all items) to 31% (“a big problem” for all items).

Among people with a recent episode of illness, perceiving no barriers in seeking medical care predicted a 41% probability of having consulted, versus a 22% probability when perceiving all four barriers as “a big problem”; conversely, the probability of self-medicating increased from 20 to 52%.

Among women with a recent pregnancy, the probability of giving birth in a health facility decreased by 37% points (i.e., from 68 to 31%) when all barriers to medical care were perceived as “not a problem” versus “a big problem”. Similarly, the predicted number of prenatal consultations was 3.69 in women with no perceived barriers, versus 2.79 for those who perceived all barriers as “a big problem”.

**Table 2:** Association of the PBMC score and primary care utilization (univariate regressions)

	**Population**	**Dependent variable**	**Model**	**Type of estimate**	**Estimate**	**p-value**	**Predictions**		
							At Score = 0 (“not a problem”)	At Score = 1 (“a small problem”)	At Score = 2 (“a big problem”)
**Primary care utilization**	All adults (n = 1,787)	Forgone medical consultation (HH level)	Logistic	OR	3.10^***^ (0.45)	< 0.001	0.20 (0.02)	0.43 (0.02)	0.70 (0.04)
		Forgone medical treatment (HH level)	Logistic	OR	1.30 (0.18)	0.061	0.21 (0.02)	0.26 (0.02)	0.31 (0.04)
	Participants with a recent episode of illness (n = 418)	Consulted in a health facility following an episode of illness	Logistic	OR	0.63^*^ (0.15)	0.047	0.41 (0.05)	0.30 (0.03)	0.22 (0.05)
		Self-medicated following an episode of illness	Logistic	OR	2.09^**^ (0.47)	0.001	0.20 (0.04)	0.34 (0.03)	0.52 (0.07)
	Women with a recent birth (n = 197)	Gave birth in a health facility	Logistic	OR	0.46^*^ (0.16)	0.023	0.68 (0.07)	0.49 (0.05)	0.31 (0.10)
		Number of prenatal consultations	Poisson	IRR	0.87^*^ (0.06)	0.028	3.69 (0.20)	3.20 (0.12)	2.78 (0.25)

Notes: *p < 0.05, **p < 0.01, ***p < 0.001. All variables measured at the individual level, unless when HH-level specified. Robust standard errors (clustered at the household level to account for intra-household correlation) in parenthesis. Regressions were weighted using sampling weights to account for choice-based stratified samples. All binary dependent variables were coded as 0 for “no” and 1 for “yes”. For logistic models, predictions are predicted probabilities of the dependent variable. For Poisson models, predictions are the predicted number of events

Abbreviations: n = number of observations, HH = household, OR = odds ratio, CBHI = community-based health insurance, IRR = incidence-rate ratio

### Confirmatory dataset

In the AmBASS dataset, items (3)-(6) (hereafter ‘reduced PBMC score’) yielded a 0.71 Cronbach’s alpha indicating good internal consistency. Stepwise descendant EFA conducted in the AmBASS dataset yielded a one-factor score with the full set of items (hereafter, ‘full PBMC score’). The CFA model estimated on items of the reduced PBMC score fitted the data well—as indicated by goodness-of-fit measures, which got worse when computed on the full set of items. Both the reduced and full PBMC scores were significantly associated with determinants and healthcare seeking (e.g., education, age, residency, resources), and primary care utilization. These results confirm the construct and predictive validity of the four-item PBMC score in the general population of the Niakhar HDSS, but suggest that the structure of the score may be sample-dependent. All details were reported in Appendix A6 in the Supplementary Material.

## Discussion

As in the 2012 study on women from Burkina Faso [23], we found that obstacles were higher in under-educated, poorer individuals and those living in rural areas (i.e., in our sample, participants living further away from semi-urban—health—facilities). In contrast, in both samples used in the present study, the EFA yielded a one-dimensional factor score, whereas Nikiema and colleagues built a second-stage score combining all six items over three dimensions (specifically, psychosocial, socioeconomic, and geographic barriers). However, the Burkina Faso data was from 2005, only among women, a sizeable share of whom was living in urban areas. This suggests that the structure of the score might need to be validated when computed in very different settings or samples.

In line with the literature, we found that perceived barriers were strongly associated with the utilization of prenatal and maternal health services [21, 38]. In our study, the PBMC score’s prediction of health services utilization was robust to the type of primary care utilization, health need, and population: specifically, the score can be employed to predict the probability of foregoing medical consultation or expenses at the household level, of medical consultation and non-utilization (self-medication) in individuals with a recent episode of illness, and of maternal health services utilization in women who had a live birth the past two years (documented through delivery in a health facility and the number of prenatal consultations).

### Value-added of the PBMC score and policy implications

The main implications for public health practitioners are two-fold. First, our results highlight the importance to pay attention to perceived obstacles, both in terms of number and intensity—as they predict primary care utilization in rural sub-Saharan Africa. Second, through the PBMC score, we offer a valid, synthetic, simple, and sensitive measure to be used in future studies.

Unlike measures of access focusing on individuals that experienced an event prompting health services utilization (e.g., individuals with a recent episode of illness or women with a recent pregnancy or birth), the PBMC score can be documented in the general population through simple, and relatively light data collection and data analysis processes.

The factor-based score also has the advantage of being expressed in the same scale as the original items, with values that can be easily interpreted: a 0 score corresponds to having declared “not a problem” to all items, a 2 score indicates that all items were reported as “a big problem”, and values in between reflect increasing levels in barriers. In contrast to studies documenting ‘any’ perceived barrier [12, 21] or focusing on a specific barrier such as distance [20], the PBMC score, therefore, provides a much more precise and sensitive measure of both the intensity and the width of barriers to medical care. Additionally, with a factor-based score, only the structure of the score (i.e. the selection of the set of items used to build the score) may be sample-dependent.

As illustrated by the absence of association with CHE, the PBMC score captures something other than financial risk protection. Indeed, our results suggest that people who perceive high financial barriers in accessing healthcare are less likely to afford or incur significant healthcare expenses. The score is therefore valuable in providing information on additional deficits in, and progress towards UHC attainment. There is a wide range of possible uses for the score. For instance, the identification of individual and structural characteristics associated with the intensity of the score can help characterize populations and areas that should be targeted by specific interventions or policies aiming at improving UHC. The score can also be used to evaluate such interventions through the comparison of changes in individual score levels over time (before/after intervention or longitudinal studies)—to name just a few potential applications.

### Limits

Our study has limitations. The main concern is that it relies on self-reported measures, which can be subject to heterogeneity in reporting associated with psycho-social and socio-economic variables—such biases have been extensively documented in the literature on self-assessed health [39–44]. In addition, our results reveal an association between the PBMC score and psychosocial variables (specifically risk aversion, generalized trust, and perceived quality of the healthcare system), which ought to be accounted for, both in potentially future multivariate regressions and in policy design. However, we provide ample evidence that our score is significantly associated with objective measures and determinants of healthcare-seeking (distance to the health facility, sex, formal education, several measures of wealth and poverty, etc.).

A second limitation is that, though multidimensional, the PBMC score only provides a partial view of access. In particular, it does not include supply-side information on the availability or quality of healthcare services, professionals, equipment, or medications in the area of interest—i.e., the health system’s side of Levesque’s comprehensive framework of patient-centered access to healthcare [45]. Items used to build the PBMC score encompasses the “ability to seek”, “ability to reach” and “ability to pay” of populations defined in this framework, but its scope falls short of abilities to perceive and engage that are instrumental in the populations’ access to healthcare.

A final, and related, limitation is that, by using DHS-based items in a top-down process, the PBMC score may overlook context-specific barriers that are relevant to accessing healthcare goods and services in rural Senegal. Bottom-up approaches to tailoring items to the specific context would gain in internal validity though potentially at the expense of external validity^3^. Indeed, the PBMC score has the ambition of being used in other settings, e.g. through DHS surveys, though data availability is limiting—especially in men^4^.

## Conclusion

We used DHS-based items on perceived barriers to medical care to build a one-dimensional score in both men and women living in rural Senegal. This PBMC score is internally consistent and confirmed in the Niakhar HDSS using a different dataset representative of adult individuals living in the same area. The score is significantly associated with a wide range of determinants of healthcare-seeking (including, but not limited to, sex, education, marital status, poverty, and distance to the health facility). Additionally, the score can predict non-utilization of primary care at the household level, utilization and non-utilization of primary care following an individual’s episode of illness, and utilization of primary care during pregnancy and birth. The score was confirmed using CFA in the general adult population living in the Niakhar HDSS, though further investigation is warranted to confirm its validity in other settings.

As a valid, sensitive, and easily documented individual-level indicator, the PBMC score can be a complement to regional or national level health services coverage to measure health services access and utilization. At the individual or household level, the PBMC score can also be combined with conventional metrics of financial risk protection such as CHE to comprehensively document deficits in, and progress towards UHC.

## Electronic supplementary material

Below is the link to the electronic supplementary material.

Supplementary Material 1

## Data Availability

Data are available from the authors upon reasonable request (contact: Marion COSTE, AMU—AMSE, 5–9 Boulevard Maurice Bourdet, CS 50,498, 13,205 Marseille Cedex 1, + 33,652,465,772, marion.coste@univ-amu.fr). The code is also available from the authors upon reasonable request.

## Footnotes

1 Specifically, the 2008 DHS model woman’s questionnaire read “*Many different factors can prevent women from getting medical advice or treatment for themselves. When you are sick and want to get medical advice or treatment, is each of the following a big problem or not a big problem?*” and offered “not a big problem” versus “a big problem” as options. The 2012 and 2020 questionnaires used a similar phrasing but only document the items “permission to go”, “getting money”, “distance” and “go alone”.

2 The reduced set was of higher internal consistency than both the original set (0.6295) and the 5-items retained after stepwise descendant EFA (0.6577).

3 See for instance the process of building a bottom-up index of well-being for women living in rural Malawi from focus groups and ordered preferences [46, 47].

4 To a lesser extent, data availability is also a concern for women: in the most recent waves of the DHS surveys, women’s questionnaires have been reduced to binary responses (“a big problem” versus “not a big problem”) for four items (permission, money, distance, and going alone).

## Abbreviations

BSF: Senegalese government family allowance (“*Bourse de Sécurité Familale*”).
CBHI: community-based health insurance.
CFA: confirmatory factor analysis.
CHE: catastrophic health expenditures.
DHS: demographic and health surveys.
EFA: explanatory factor analysis.
HDSS: health and demographic surveillance system.
LMICs: low and middle-income countries.
PBMC: perceived barriers to medical care.
OOP: out-of-pocket (health expenditures).
UHC: universal health coverage.
WHO: World Health Organization.
