# Supplementary material for: Predicting health services utilization using a score of perceived barriers to medical care: evidence from rural Senegal
Source: BMC Health Serv Res. 2023 Mar 16;23:263. doi: 10.1186/s12913-023-09192-2 (PMC10018867; doi:10.1186/s12913-023-09192-2)
Supplement: Supplementary file 1 — Supplementary Material 1 [file 12913_2023_9192_MOESM1_ESM.docx]

**Supplementary material**

This online resource has been provided by the authors to give readers additional information about their work.

Supplement to: **Predicting health services utilization using a score of perceived barriers to medical care: evidence from rural Senegal**

**Contents:**

**Page 2:** Appendix A1. Members of the UNISSAHEL Study Group

**Page 3-4:** Appendix A2. Definitions of all variables used

**Page 5-6:** Appendix A3. Results of the explanatory factor analysis

**Page 7:** Appendix A4. Summary statistics

**Page 8-11:** Appendix A5. Predictive validity of the score: graphical representation of the univariate regression results

**Page 12-16:** Appendix A6. Confirmation of the score on the ANRS12356 AmBASS dataset

**Appendix A1. Members of the UNISSAHEL Study Group**

**Project coordination:** MP. BA (UGB), E. BONNET (RESILIENCE, IRD), F. CHABROL (CEPED, IRD), A. DESGREES DU LOU (CEPED, IRD), A. DUMONT (CEPED, IRD), F. EBOKO (CEPED, IRD), A. FILLOL (CEPED, IRD), D. MANOUFI (BASE), V. RIDDE (CEPED, IRD), K. SOW (CRCF), L. TOURE (MISELI), B. VENTELOU (AMSE, CNRS).

**Project members:** Mohammad ABU ZAINEH (AMSE), Malick Ousman AHMAT (BASE), Sameera AWAWDA (AMSE, IRD), El Hadj BA (VITROME, IRD), Pauline BOIVIN (MISELI), Marwân-al-Qays BOUSMAH (CEPED, IRD), Sylvie BOYER (SESSTIM, Aix-Marseille Université), Valérie DELAUNAY (LPED, IRD), Fatimatou DIA (UGB), Mariama DIEDHIOU (CRCF), Rama Sao DIOP (UGB), Fatou DIOP (CRCF), Cheikh Mouhamadou Falilou Mb. FALL (UGB), Laurence FLEURY (LPED, IRD), Idriss Ali Zakaria GALI-GALI (BASE), Lara GAUTIER (UDEM), Wendyam Charles Paulin Didier KABORE (CEPED, IRD), Yamba KAFANDO (independent consultant), Richard LALOU (MERIT, IRD), Ramadan KAGONTA (BASE), Gabrièle LABORDE-BALEN (TRANSVIHMI, IRD), Fatou Bintou NIANG (UGB), Ratnan NGADOUM (Université de N’Djamena), Khady Seck NGOM (CRCF), El Hadji Ibrahima Kady SARR (UGB), Mathieu SEPPEY (UDEM), Cheikh SOKHNA (VITROME, IRD), Souleymane SOW (CRCF), Bernard TAVERNE (TRANSVIHMI, IRD), Cheikh TINE (MERIT, IRD), Grâce-à-Dieu TOULAO (IRD), Bintou RASSOUL TOP (CRCF), Mamadou YATOUDEME (IRD).

**Acronyms:** AMSE (France): Aix-Marseille School of Economics, CEPED (France): Centre Population et Développement, CNRS (France): Centre National de la Recherche Scientifique, CRCF (Senegal): Centre Régional de Recherche et de Formation à la Prise en Charge Clinique de Fann, LASDEL (Niger): Laboratoire d'Etudes et de Recherche sur les Dynamiques Sociales et le Développement Local, LPED (France): Laboratoire Population Environnement Développement, SESSTIM (France): Sciences économiques et sociales de la santé et traitement de l’information médicale, UDEM (Canada): Université de Montréal, UGB (Sénégal): Université Gaston Berger de Saint Louis.

**Appendix A2. Definitions of all variables used**

| Variable group | Variable | Type | Definition | N | % |
| --- | --- | --- | --- | --- | --- |
|  |  |  |  |  |  |
| Determi-nants of healthcare-seeking | Had primary education or higher | Binary | None (=reference category); Primary school or higher | 1787 | 83.77; 16.23 |
|  | Was a woman | Binary | Man (=reference category); Woman | 1787 | 45.89; 54.11 |
|  | Was in a union | Binary | Not in a union (=reference category); In a union | 1787 | 9.78; 90.22 |
|  | Age | Continuous | Age (in years) | 1787 |  |
|  | Was poor (monetary poverty, HH level) | Binary | Household not considered as poor based on a monetary poverty indicator (=reference category); Considered as poor | 1787 | 49.09; 50.91 |
|  | Was poor (food poverty, HH level) | Binary | Household not considered as poor based on a food poverty indicator (=reference category); Considered as poor | 1787 | 61.87; 38.13 |
|  | Was poor (subjective poverty, HH level) | Binary | Household not considered as poor based on a subjective poverty indicator (=reference category); Considered as poor | 1787 | 70.84; 29.16 |
|  | Monthly consumption expenditures per adult equivalent (in CFA francs) | Continuous | Total monthly consumption expenditures per adult equivalent in the household | 1787 |  |
|  | Number of adult equivalents in the household (HH level) | Continuous | Number of adult equivalents in the household, calculated using the Food and Agriculture Organization (FAO)’s Adult Male Equivalent (AME) method | 1787 |  |
|  | Distance to the nearest healthcare facility (in km) | Continuous | Shortest geographical distance (in km) between the household and the nearest healthcare facility (based on GPS coordinates) | 1787 |  |
|  | Distance to the nearest CBHI (in km) | Continuous | Shortest geographical distance (in km) between the household and the nearest CBHI (based on GPS coordinates) | 1787 |  |
| Other potentially-associated variables | Had an at least fair knowledge of CBHI | Binary | Never heard of CBHI schemes/No knowledge (=reference category); Fair/good knowledge | 1787 | 73.88; 26.12 |
|  | Health insurance status | Polytomous | Not enrolled in a CBHI (=reference category); Enrolled (voluntarily); Enrolled (subsidized) | 1787 | 87.38; 4.11; 8.51 |
|  | Willingness to pay for CBHI (in CFA francs) | Continuous | Maximum annual premium an individual would pay to enroll in a CBHI (in CFA francs) | 1787 |  |
|  | Had a chronic illness | Binary | No chronic illness (=reference category); Chronic illness | 1787 | 91.16; 8.84 |
|  | Had a handicap | Binary | No handicap (=reference category); Handicap | 1787 | 95.04; 4.96 |
|  | Had a poorer health | Binary | Excellent/Very good health (=reference category); Good/Fair/Poor health | 1787 | 41.52; 58.48 |
|  | SF-12 Mental Component Summary (MCS-12) | Continuous | Mental health score calculated from the SF-12 questionnaire, ranging from 0 to 100 (higher values corresponding to better health-related quality of life) | 1787 |  |
|  | SF-12 Physical Component Summary (PCS-12) | Continuous | Physical health score calculated from the SF-12 questionnaire, ranging from 0 to 100 (higher values corresponding to better health-related quality of life) | 1787 |  |
|  | Perception of healthcare quality | Continuous | Factor-based score (higher values corresponding to a lower perception of healthcare quality), encompassing nine dimensions about the health facility the most frequently visited by the respondent (the premises, the medical material and equipment, the waiting time, the physician’s listening skills, the physical examination, the medical care provided, the medical staff guidance, the reliability of the diagnosis, and the availability of drugs) | 1787 |  |
|  | Risk tolerance | Discrete | Qualitative scale ranging from 0 (“not at all willing to take risks”) to 10 (“very willing to take risks”) | 1785 |  |
|  | Generalized trust | Discrete | Qualitative scale ranging from 0 (“you can’t be too careful in dealing with people”) to 10 (“most people can be trusted”) | 1786 |  |
| Catastro-phic health expendi-ture | Had catastrophic health expenditures, 40% threshold (HH level) | Binary | The household's out-of-pocket health expenditure did not exceed 40% of its capacity to pay (=reference category); The household's out-of-pocket health expenditure exceeded 40% of its capacity to pay | 1787 | 93.68; 6.32 |
|  | Had catastrophic health expenditures, 30% threshold (HH level) | Binary | The household's out-of-pocket health expenditure did not exceed 30% of its capacity to pay (=reference category); The household's out-of-pocket health expenditure exceeded 30% of its capacity to pay | 1787 | 89.30; 10.70 |
|  | Had catastrophic health expenditures, 20% threshold (HH level) | Binary | The household's out-of-pocket health expenditure did not exceed 20% of its capacity to pay (=reference category); The household's out-of-pocket health expenditure exceeded 20% of its capacity to pay | 1787 | 82.82; 17.18 |
| Primary care utilization | Forgone medical consultation (HH level) | Binary | The household did not have to forgo medical consultation in the last 12 months due to financial hardship (=reference category); The household forgone medical consultation | 1787 | 64.57; 35.43 |
|  | Forgone medical treatment (HH level) | Binary | The household did not have to forgo medical treatment in the last 12 months due to financial hardship (=reference category); The household forgone medical treatment | 1787 | 75.85; 24.15 |
|  | Consulted in a health facility following an episode of illness | Binary | Did not consult in a health facility in case of health problem that occurred in the last 2 months (=reference category); Consulted | 418 | 67.38; 32.62 |
|  | Self-medicated following an episode of illness | Binary | Did not self-medicate in case of health problem that occurred in the last 2 months (=reference category); Self-medicated | 418 | 68.73; 31.27 |
|  | Gave birth in a health facility | Binary | Delivered at home, for a live birth that occurred in the last 2 years (=reference category); Delivered in a health facility | 197 | 45.81; 54.19 |
|  | Number of prenatal consultations | Discrete | Number of prenatal consultations (for a live birth that occurred in the last 2 years) | 197 |  |
| Notes: All variables measured at the individual level, unless when HH-level specified. Data were weighted using sampling weights to account for choice-based stratified samples.Abbreviations: N=number of observations, %=percentage of populations or subpopulations (for binary and polytomous variables), HH=household, CBHI=community-based health insurance. | | | | | |

**Appendix A3. Results of the explanatory factor analysis**

Factor analysis/correlation Number of obs = 1,787

Method: principal-component factors Retained factors = 2

Rotation: orthogonal varimax (Kaiser off) Number of params = 9

Factor Variance Difference Proportion Cumulative

Factor1 **2.13304** 1.08438 **0.4266**  0.4266

Factor2 1.04866 . 0.2097 0.6363

LR test: independent vs. saturated: chi2(10) = 2035.55 Prob>chi2 = 0.0000

Rotated factor loadings (pattern matrix) and unique variances

Variable Factor 1 Factor 2 Uniqueness

v2_A9_Q3_B 0.0775 0.8906 0.2008

v2_A9_Q3_C **0.5390** -0.3635 0.5773

v2_A9_Q3_D **0.8768** 0.0606 0.2276

v2_A9_Q3_E **0.8852** 0.0748 0.2108

v2_A9_Q3_F **0.5331** 0.3376 0.6018

**Figure A3.1. Scree plot**


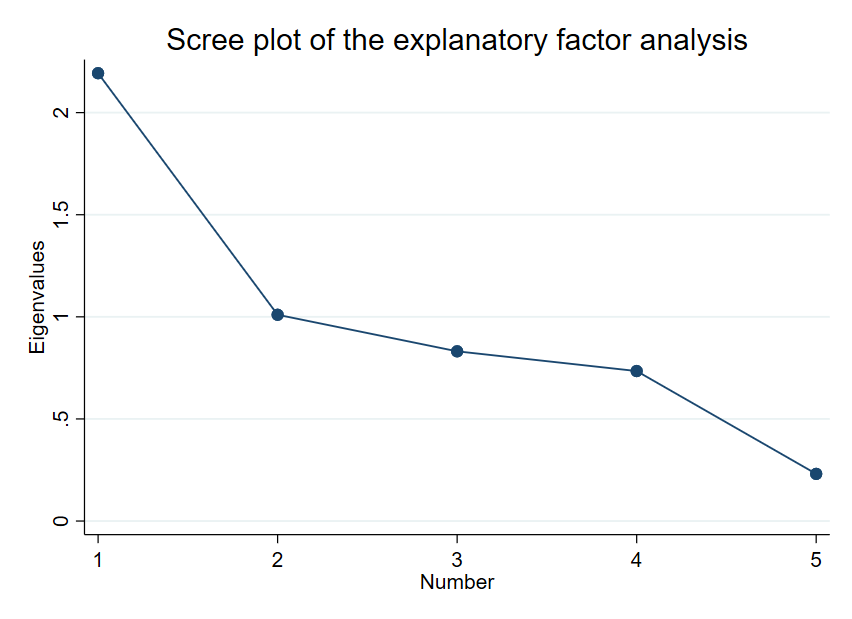


We retained factor 1 (eigenvalue = 2.13 > 1.1), which was situated right before the “elbow” of the scree plot (see Figure A2.1).

**Figure A3.2 Density of the 4-item PBMC score**

**Appendix A4. Summary statistics**

| Variable group | Variable | Type | Mean or proportion | Standard deviation | Min | Max |
| --- | --- | --- | --- | --- | --- | --- |
| Determinants of healthcare-seeking | Had primary education or higher | Binary | 0.16 | 0.37 | 0.00 | 1.00 |
|  | Was a woman | Binary | 0.54 | 0.50 | 0.00 | 1.00 |
|  | Was in a union | Binary | 0.90 | 0.30 | 0.00 | 1.00 |
|  | Age | Continuous | 52.85 | 13.85 | 15.00 | 94.00 |
|  | Was poor (monetary poverty, HH level) | Binary | 0.51 | 0.50 | 0.00 | 1.00 |
|  | Was poor (food poverty, HH level) | Binary | 0.38 | 0.49 | 0.00 | 1.00 |
|  | Was poor (subjective poverty, HH level) | Binary | 0.29 | 0.45 | 0.00 | 1.00 |
|  | Monthly consumption expenditures per adult equivalent (in CFA francs) | Continuous | 16,936.27 | 10,887.67 | 2,868.00 | 162887.59 |
|  | Number of adult equivalents in the household (HH level) | Continuous | 11.53 | 5.94 | 0.79 | 41.90 |
|  | Distance to the nearest healthcare facility (in km) | Continuous | 3.15 | 2.19 | 0.01 | 9.40 |
|  | Distance to the nearest CBHI (in km) | Continuous | 5.46 | 2.84 | 0.05 | 12.82 |
| Other potentially-associated variables | Had an at least fair knowledge of CBHI | Binary | 0.26 | 0.44 | 0.00 | 1.00 |
|  | Willingness to pay for CBHI (in CFA francs) | Continuous | 3,865.83 | 3,905.57 | 0.00 | 50,000.00 |
|  | Had a chronic illness | Binary | 0.09 | 0.28 | 0.00 | 1.00 |
|  | Had a handicap | Binary | 0.05 | 0.22 | 0.00 | 1.00 |
|  | Had a poorer health | Binary | 0.58 | 0.49 | 0.00 | 1.00 |
|  | SF-12 Mental Component Summary (MCS-12) | Continuous | 47.89 | 7.81 | 18.92 | 70.56 |
|  | SF-12 Physical Component Summary (PCS-12) | Continuous | 49.53 | 9.52 | 17.51 | 65.27 |
|  | Perception of healthcare quality | Continuous | 0.52 | 0.53 | 0.00 | 2.56 |
|  | Risk tolerance | Discrete | 5.18 | 2.48 | 0.00 | 10.00 |
|  | Generalized trust | Discrete | 5.20 | 2.23 | 0.00 | 10.00 |
| Catastrophic health expenditure | Had catastrophic health expenditures, 40% threshold (HH level) | Binary | 0.06 | 0.24 | 0.00 | 1.00 |
|  | Had catastrophic health expenditures, 30% threshold (HH level) | Binary | 0.11 | 0.31 | 0.00 | 1.00 |
|  | Had catastrophic health expenditures, 20% threshold (HH level) | Binary | 0.17 | 0.38 | 0.00 | 1.00 |
| Primary care utilization | Forgone medical consultation (HH level) | Binary | 0.35 | 0.48 | 0.00 | 1.00 |
|  | Forgone medical treatment (HH level) | Binary | 0.24 | 0.43 | 0.00 | 1.00 |
|  | Consulted in a health facility following an episode of illness | Binary | 0.33 | 0.47 | 0.00 | 1.00 |
|  | Self-medicated following an episode of illness | Binary | 0.31 | 0.46 | 0.00 | 1.00 |
|  | Gave birth in a health facility | Binary | 0.54 | 0.50 | 0.00 | 1.00 |
|  | Number of prenatal consultations | Discrete | 3.33 | 1.26 | 0.00 | 6.00 |
| Notes: Means were computed for continuous and discrete variables, and proportions were computed for binary variables. All variables measured at the individual level, unless when HH-level specified. Data were weighted using sampling weights to account for choice-based stratified samples.Abbreviations: N=number of observations, HH=household, CBHI=community-based health insurance. | | | | | | |

**Appendix A5. Predictive validity of the score: graphical representation of the univariate regression results**

**Appendix A5.1 PBMC score and determinants of healthcare-seeking**

**Appendix A5.2 PBMC score and other variables of interest**

**Appendix A5.3 PBMC score and catastrophic health expenditures**

**Appendix A5.4 PBMC score and primary care utilization and non-utilization**


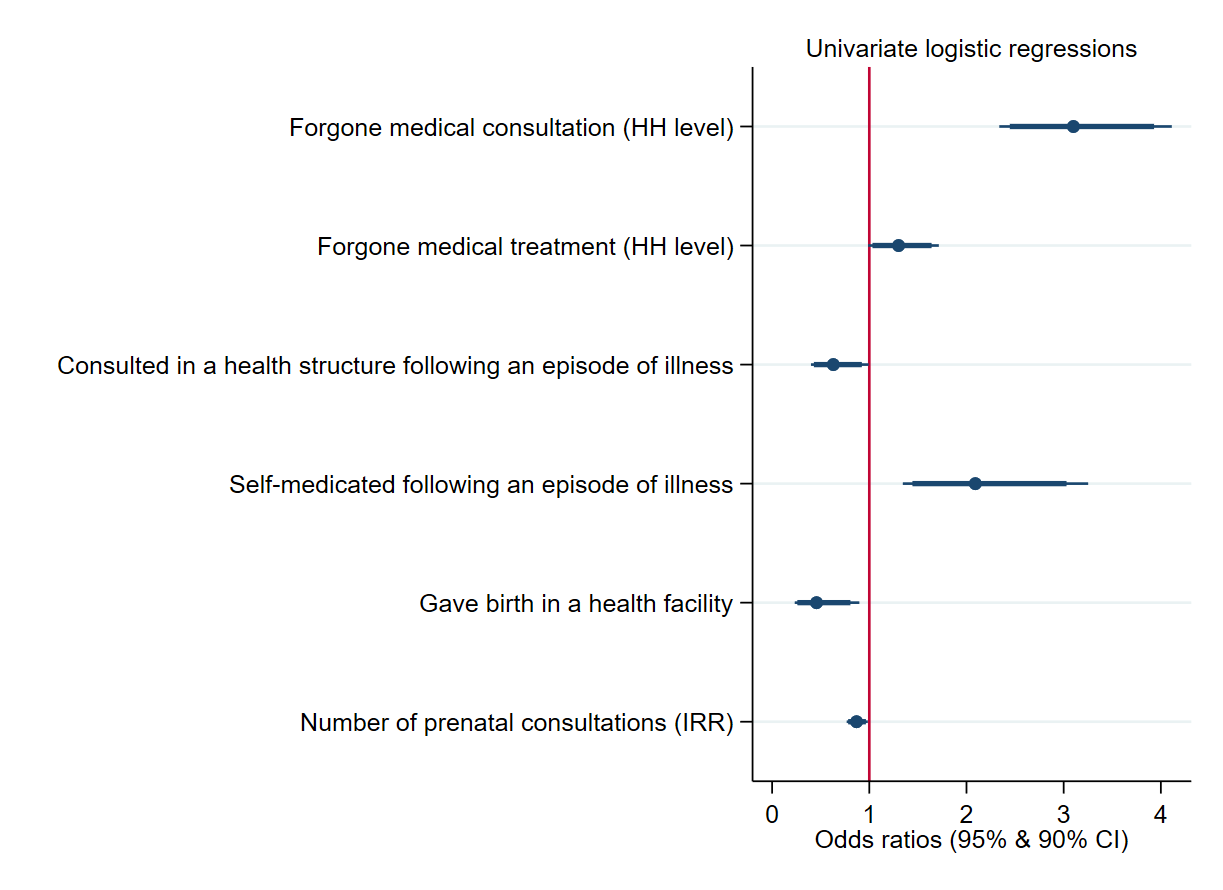


**Appendix A6. Confirmation of the score on the ANRS12356 AmBASS dataset**

The AmBASS survey was conducted between October 2018 and July 2019 in 12 villages of the Niakhar HDSS. Households were randomly selected, and all their residents (over 6 months of age) were invited to participate, ensuring a sample representative of people living in the Niakhar area. DHS-based items were administered to all 724 participants, age 15 and above, interviewed between January and July 2019.

1. **Perceived barriers to medical care in the AmBASS dataset**

Compared with the CMUtuelleS dataset (see Figure A6.1.), a smaller share of participants reported having money, distance, and finding transport as a ‘big problem’ (11% vs. 55%, 5% vs. 15%, and 2% vs. 10%, respectively), and a bigger share of participants identified knowing where to go and getting the permission to go as ‘a small problem’ (13% vs. 2% and 12% vs. 1%, respectively).


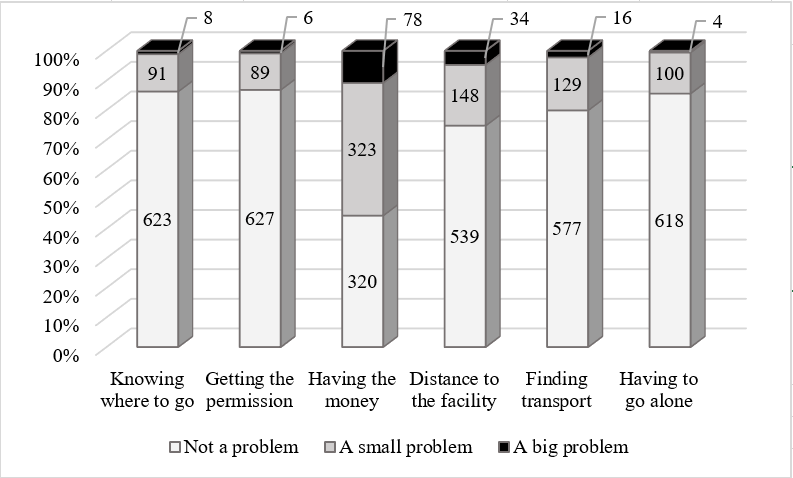


**Figure A6.1.** Perceived barriers to medical care (items considered for the score)

1. **Confirmatory factor analysis**

Table A6.1 presents results from confirmatory factor analysis on the AmBASS dataset.

| **CFA models** | | **Chi2 (p-value)** | **RMSEA** | **SRMR** | **CFI** | **TLI** |
| --- | --- | --- | --- | --- | --- | --- |
| (1) | AmBASS: reduced | 5.49 (0.0642) | 0.049 | 0.016 | 0.996 | 0.988 |
| (2) | AmBASS: full | 241.943 (0.00) | 0.190 | 0.064 | 0.860 | 0.767 |
| (3) | AmBASS: full with co-variates | 18.967 (0.00) | 0.055 | 0.019 | 0.992 | 0.981 |

**Table A6.1.** Goodness of fit measures of the CFA analysis

We first conducted CFA on the ‘reduced’ set of items validated in the CMUtuelleS survey, specifically items (3), (4), (5), and (6). Goodness of fit measures indicated an excellent fit of the data, better than when including the full set of items, and even after including co-variates (models (2) and (3) respectively).

**3. Building the score in the AmBASS dataset**

Our sample passed the Bartlett test of sphericity, rejecting the null hypothesis that variables are not inter-correlated (γ²=1674.965(15), p-value=0.000), and gave a value for the Kaiser-Meyer-Olkin measure sufficiently large (0.807) to justify running a factor analysis. Stepwise descendant factor analysis showed that removing any of the items would significantly reduce the quality of the factor analysis. Subsequent factor analysis was therefore conducted on all 6 items. Following EFA and scree plot analysis, only one dimension was retained (3.28 eigenvalue, explaining 55% of variations, see Figure A6.2).

**Figure A6.2** Exploratory factor & scree plot analysis

Rotations with weights revealed that all items significantly contributed to dimension one (loadings > 0.4). The 0.79 Cronbach’s alpha indicated excellent internal consistency. We, therefore, built a factor-based score with the average of items (1)-(6) on individuals without missing values (n=720). For comparison and cross-validation purposes, we also built a reduced score as the average of items (3)-(6). This reduced score exhibited good internal consistency with a 0.71 Cronbach’s alpha. Table A6.2 presents the score (bar charts are also provided in Figure A6.3).

| **Score** | **Items** | **Mean (min;max)** | **Median [IQR]** | **Cronbach’s α** |
| --- | --- | --- | --- | --- |
| Reduced PBMC | (3)-(6) | 0.33(0;2) | 0.25[0-1] | 0.71 |
| Full PBMC | (1)-(6) | 0.27(0;2) | 0.17[0-0.33] | 0.79 |

**Table A6.2.** Summary statistics and internal consistency of the full and reduced PBMC scores

**

Reduced BMC score Full PBMC score

**Figure A6.3** Full and reduced PBMC scores

Table A6.3 displays the results of the univariate regressions of the score on similar variables than in the main analysis. Results suggested that the choice of items can be sample dependent, but had no impact on the validity of the score (i.e., no significant differences between reduced and full PBMC scores). In conclusion, the PMBC score was valid in the general population of the Niakhar area.

**Table A6.3**. Univariate regressions of the full and reduced PBMC score

| Variable group | Dependent variable | Model | Type of estimate | Estimates | | N |
| --- | --- | --- | --- | --- | --- | --- |
|  |  |  |  | Reduced set | Full set |  |
| Determinants of healthcare seeking | Had primary education or higher | Logistic | OR | 0.34^**^(0.73) | 0.34^**^(0.09) | 711 |
|  | Being a woman | Logistic | OR | 1.45(0.28) | 1.42(0.31) | 720 |
|  | Was in a union | Logistic | OR | 1.65^*^(0.42) | 1.50(0.45) | 720 |
|  | Age | Linear | CE | 5.69^**^(1.77) | 5.00^*^(2.00) | 720 |
|  | Over 20 years of age | Logistic | OR | 1.78^*^(0.49) | 1.75^*^(0.60) | 720 |
|  | Living in a semi-urban village | Logistic | OR | 0.20^**^(0.06) | 0.19^**^(0.07) | 720 |
|  | Living conditions index | Linear | CE | -0.53^**^(0.13) | -0.57^**^(0.15) | 720 |
|  | Agricultural resources index | Linear | CE | 0.39^**^(0.15) | 0.47^**^(0.18) | 720 |
|  | Number of adults in the household | Linear | CE | -1.13^*^(0.57) | -1.29(0.69) | 720 |
|  | Monetary resources (HH level) | Linear | CE | -1650719^*^ (774846.6) | -2017058^**^ (936073.9) | 720 |
| Other potentially-associated variables | Ever heard of hepatitis B | Logistic | OR | 0.44^*^(0.15) | 0.38^*^(0.16) | 720 |
|  | Covered by insurance | Logistic | OR | 0.04^**^(0.04) | 0.007^**^(0.013) | 720 |
|  | SF-12 Mental Component Summary (MCS-12) | Linear | CE | 4.44^**^(1.21) | 6.35^**^(1.39) | 699 |
|  | SF-12 Physical Component Summary (PCS-12) | Linear | CE | -5.46^**^(0.75) | -6.20^**^(0.84) | 699 |
| Primary care utilization | Consulted a shaman during a recent episode of illness | Logistic | OR | 29.03^**^(17.30) | 19.64^**^(18.22) | 102 |
|  | Place of birth delivery (last pregnancy)Ref=at home | Multinomial logistic | RRR (dispensary) | -0.37(0.42) | -0.49(0.49) | 180 |
|  |  |  | RRR (medicalized) | -2.06^*^(0.84) | -2.95^**^(1.06) | 180 |
| Notes: ^∗^ p < 0.05, ^∗∗^ p < 0.01. All variables measured at the individual level, unless when HH-level specified. Robust standard errors (clustered at the household level to account for intra-household correlation) in parenthesis. Regressions were weighted using sampling weights to account for choice-based stratified samples. All binary dependent variables were coded as 0 for “no” and 1 for “yes”.Abbreviations: N=number of observations, HH=household, OR=odds ratio, CE=coefficient estimate, RRR=relative-risk ratio. | | | | | | |
